# Supplementary material for: WordCommentsAnalyzer: A windows software tool for qualitative research
Source: F1000Res. 2018 Sep 4;7:536. Originally published 2018 May 3. [Version 2] doi: 10.12688/f1000research.14819.2 (PMC6137411; doi:10.12688/f1000research.14819.2)
Supplement: Supplementary file 3 [file f1000research-7-17614-s0002.tgz › b2a79496-4ada-47c4-9682-a99ce867836d.zip!/Eight Iranian Health Journals/Archives of Rehabilitation_55.docx]

© Archives of Rehabilitation. This work is licensed under CC BY-NC 4.0 (https://creativecommons.org/licenses/by-nc/4.0/).

Volume & Issue: دوره 7 - شماره 1

Title: بهبود عملکرد حرکتی نخاع به‌دنبال پیوند سلول‌های استرومای مغز استخوان در موش صحرایی

http://rehabilitationj.uswr.ac.ir/article-1-54-fa.html

هدف: یافتن راههای درمانی مؤثر برای آسیب‌های سیستم عصبی همواره یکی از مهمترین اهداف دانشمندان علوم اعصاب بوده است. در پژوهش حاضر اثر پیوند سلولهای استرومایی مغز استخوان (BMSC) با استفاده از ژل فوم در بهبود حرکتی موش صحرایی بالغ، ‌پس از قطع نیمه نخاع در سطح مهره 13T بررسی شد. روش بررسی: پنج گروه موش صحرایی بالغ با انتخاب ساده تصادفی و به روش تجربی و مداخله‌ای مورد بررسی قرار گرفتند. در نخاع گروه اول یا کنترل هیچ دستکاری صورت نگرفت. در گروه های دوم تا پنجم نیمی از نخاع قطع شد، در گروه دوم هیچ درمانی انجام نشد، ‌در گروه سوم ماده ژل فوم حاوی محیط کشت به محل قطع پیوند شد، در گروه چهارم علاوه بر پیوند ژل فوم سلولهای BMSC هم به محل قطع تزریق گردید و در گروه پنجم ژل فوم آغشته به BMSC پیوند زده شد. سلولهای BMSC از مغز استخوان موش صحرایی به دست آمد. در همه گروه‌ها در روز اول و در پایـان هـفته‌های اول تا پنجم بعد از جراحی تـست حرکتی BBB انجام شد و پس از پایان هفته پنجم قطعات نخاعی6L - 4L برای مطالعات شمارش سلولی مورد استفاده قرارگرفت. یافته‌ها: میانگین نمره تست حرکتی در گروه کنترل هیچ تغییری نداشت ولی میانگین نمرات این تستها در گروههای دوم تا پنجم با گذشت زمان افزایش داشت. همچنین نتیجه شمارش نرونهای حرکتی در نیمه قطع شده نخاع نسبت به نیمه سالم نشانگر کاهشی برابر با 0% در گروه اول، %6/64 در گروه دوم، %6/64 در گروه سوم، %9/47 در گروه چهارم و %5/53 در گروه پنجم بود. نتایج کمی هردو روش (تست حرکتی و شمارش سلولی) اختلاف معناداری را بین گروههای چهارم و پنجم با دیگر گروه‌ها نشان داد که مؤید نقش مؤثر BMSC در بهبود عملکرد نخاع است (P>0/05 و P>0/01). عدم اختلافی معنادار میان گروه‌های دوم و سوم نشان داد که ژل فوم به تنهایی نمی‌تواند در بهبود عملکرد حرکتی نخاع مؤثر باشد. نتیجه‌گیری: می‌توان نتیجه گرفت که سلول‌های B‏MSC با استقرار در محل ضایعه موجب بهبود عملکرد حرکتی نخاع می‌شوند و ژل فوم پیوندی نیز داربستی را در محل ضایعه می‌سازد که سلولهای BMSC روی آن مستقر می‌شوند.

Title: مقایسه وضعیت و راستای قرار گیری سه‌بعدی کتف در بیماران مبتلا به سندرم گیرافتادگی شانه با افراد سالم

http://rehabilitationj.uswr.ac.ir/article-1-55-fa.html

هدف: سندرم گیرافتادگی از جمله شایعترین اختلالات شانه می‌باشد که 65%-44% کلیه موارد دردهای شانه را شامل می‌شود. این عارضه به دلایل مختلفی ایجاد می‌شود. تغییر شکل‌های آناتومیکی قوس کوراکواکرومیون یا سر استخوان بازو، ضعف یا فرسایش تاندونهای روتیتورکاف، سفتی کپسول خلفی، تغییر کینماتیک شانه و تغییرات وضعیتی و کنترل حرکتی نامناسب کتف از جمله عوامل ایجاد کننده این سندرم می‌باشد. هدف این مطالعه بررسی مقایسه‌ای وضعیت و راستای سه بعدی کتف در بیماران مبتلا به سندرم گیرافتادگی و افراد سالم در حین بالا بردن بازو در صفحه استخوان کتف می‌باشد. روش بررسی: در این مطالعه تحلیلی (مقایسه‌ای) که به صورت مورد – شاهد انجام شد 17 بیمار مبتلا به سندرم گیرافتادگی با میانگین سنی 82/43 سال و 17 فرد سالم با میانگین سنی 05/44 سال که به روش نمونه‌گیری غیر احتمال ساده انتخاب شده بودند مورد مقایسه قرار گرفتند. دستگاه آنالیز حرکتی (Kinemetrix) برای اندازه‌گیری جهت قرارگیری (زوایای چرخش به سمت بالا، تیلت خلفی و چرخش داخلی کتف) و وضعیت کتف (وضعیت بالا، پایین و داخلی و خارجی کتف) در زوایای بازو در کنار بدن و ابداکشن ْ45، ْ90، ْ120 و دامنه حرکتی کامل در صفحه استخوان کتف مورد استفاده قرار گرفت.  یافته‌ها: زاویه چرخش بسمت بالای کتف در زوایای ْ90 و ْ120 در گروه بیمار کاهش معنی‌داری را نسبت به افراد سالم نشان داد (P<0/05). نتیجه‌گیری: نتایج این مطالعه نشان می‌دهد که تغییر کینماتیک استخوان کتف جنبه مهمی در بیماران مبتلا به سندرم گیرافتادگی می‌باشد. کاهش چرخش بسمت بالا و تیلت خلفی کتف که در این بیماران دیده شده بایستی در توانبخشی بیماران مبتلا به سندرم گیرافتادگی در نظر گرفته شود.

Title: مقایسه وضعیت تعادل کودکان مبتلا به فلج مغزی اسپاستیک قبل و پس از یک برنامه ورزش‌درمانی

http://rehabilitationj.uswr.ac.ir/article-1-56-fa.html

هدف: هدف از تحقیق حاضر بررسی دقیق تعادل دینامیکی به مفهوم میزان نوسانات مرکز ثقل بدن از مرکز سطح اتکا در کودکان مبتلا به فلج مغزی اسپاستیک قبل و بعد از برنامه ورزش درمانی می‌باشد. روش بررسی: این پژوهش به روش شبه تجربی و مداخله‌ای و بصورت قبل و بعد بر روی تعداد 10 نفر از کودکان 15-8 ساله مبتلا به فلج مغزی اسپاستیک از نوع دی‌پلژی که با انتخاب تصادفی از نمونه‌های در دسترس انتخاب شده بودند انجام گرفت. بیماران به مدت 12 هفته تحت برنامه ورزش‎درمانی قرار گرفتند. با استفاده از دستگاه تعادل‎سنج بایودکس نوسانات مرکز ثقل آزمودنیها در حالت‎های پایدار، نیمه ‎پایدار و ناپایدار سطح اتکا و نیز با کفش و بدون کفش قبل و پس از دوره تمرینی اندازه‌گیری و مقایسه شدند. برای تحلیل آماری از اندازه‌های مکرر استفاده شد. یافته‌ها: نتایج نشان دادند که در مجموع آزمونها میانگین نوسانات مرکز ثقل در بعد از ورزش درمانی بمیزان 0/2 درجه کاهش یافته بود (P=0/001). بیشترین بهبودی در حالت نیمه‎پایدار و ناپایدار سطح اتکا و در جهت قدامی- خلفی بود. با پوشیدن کفش نوسانات بدن بیماران بطور معنی داری کاهش یافت. نتیجه‌گیری: ورزش درمانی عملکرد تعادلی بیماران فلج مغزی دی‌پلژی را بهبود می‌بخشد. عملکرد سیستم حسی عمقی لگن و تنه با ورزش درمانی ارتقا می یابد. پوشیدن کفش عامل مهمی در حفظ تعادل دینامیکی بیماران تعادلی محسوب می‌شود و می بایستی در برنامه توانبخشی این بیماران مورد توجه قرار گیرد.

Title: بررسی تأثیر پهنای بریس کانترفورس بر میزان دامنه حرکتی، درد، قدرت گرفتن و قدرت اکستانسورهای مچ دست در افراد سالم و افراد مبتلا به سندرم آرنج تنیس‌بازان

http://rehabilitationj.uswr.ac.ir/article-1-57-fa.html

هدف: بریس کانترفورس در درمان و توانبخشی التهاب اپی‌کندیل خارجی استخوان بازو (آرنج تنیس بازان) ازطریق کاهش نیروهای اضافی برروی منشاء عضلات اکستانسور و ایجاد یک منشاء عضلانی جدید تأثیر بالینی مفیدی دارد. با این وجود گزارشهای دقیقی در مورد پهنای مناسب آن در دسترس نمی‌باشد، لذا هدف ازاین مطالعه بدست آوردن یک پهنای مناسب و بررسی تأثیر پهنای بریس کانترفورس برروی دامنه حرکتی فلکسیون واکستانسیون مچ، قدرت گرفتن دست، قدرت اکستانسیون مچ و میزان درد می‌‌باشد. روش بررسی: پژوهش حاضربه روش شبه تجربی و ازنوع اندازه‌گیریهای مکرر انجام شد. دراین مطالعه 18 نفر افراد سالم بدون هیچ ضایعه عصبی- عضلانی واستخوانی دراندام فوقانی و 18 نفر مبتلا به سندرم آرنج تنیس بازان مورد بررسی قرار گرفتند. روش نمونه گیری ازنوع نمونه گیری غیر احتمالی و به روش ساده انجام گردید. دامنه حرکتی فلکسیون واکستانسیون مچ، قدرت گرفتن دست، قدرت اکستانسیون مچ و میزان درد ابتدا بدون بریس و سپس بابستن بریسهای با پهنای 1،2 و3 اینچی کانترفورس مورد ارزیابی قرار گرفت. درانتهانتایج با استفاده از اندازه‌های مکرر و آزمون تی زوج موردبررسی قرارگرفت. یافته‌ها: 1- بریس کانترفورس باهرسه پهنا باعث کاهش دامنه حرکتی مچ درافراد سالم شده ولی در مبتلایان به اپی‌کندیلیت خارجی (تنیس البو) تغییر معناداری ایجاد نکرد 2- بریس 1 اینچی کانترفورس باعث کاهش قدرت گرفتن درافراد سالم شده درحالیکه درافراد مبتلا به آرنج تنیس بازان (تنیس‌البو) بریس 2 و3 اینچی باعث افزایش قدرت گرفتن بدون دردگردید. 3- بریسهای 1، 2 و 3 اینچی کانترفورس درافراد سالم باعث کاهش قدرت اکستانسیون مچ دست و درافراد مبتلا به سندرم آرنج تنیس بازان باعث افزایش قدرت اکستانسیون بدون درد شد. 4- بریس کانترفورس باعث کاهش میزان درد در حین گرفتن شد و دراین میان بریسهای 2 و3 اینچی تأثیر بیشتری داشتند. نتیجه‌گیری: بطور کلی نتایج این پژوهش نشان داد که استفاده از بریس کانترفورس باعث افزایش قدرت گرفتن دست و اکستانسیون مچ و کاهش میزان درد درحین گرفتن در بیماران مبتلا به سندرم آرنج تنیس بازان می‌گردد. همچنین بریس 2 و 3 اینچی کانترفورس در کاهش میزان درد نسبت به بریس 1 اینچی مؤثر‌تر واقع شد.

Title: بررسی مقادیر طبیعی و تکرارپذیری آستانه درک جریان الکتریکی در فیبرهای حسی

http://rehabilitationj.uswr.ac.ir/article-1-58-fa.html

هدف: روشهای رایج الکترودیاگنوز (NCS/EMG) محدودیتهایی در تشخیص برخـی از نوروپاتی‌ها مانند نوروپاتی‌هـــای سیستـــم اتونـــوم، مراحــل حاد نوروپاتی‌ها (hyperesthetic) و رادیکولوپاتی‌های صرفاً حسی دارند. در این زمینه روشهای بررسی کمی (Quantitative) سیستم عصبی می‌تواند جایگزین مناسبی برای روشهای رایج باشد. روش تعیین آستانه درک جریان (CPT) یکی از همین روشهای کمی است که در آن از تحریکات الکتریکی استفاده می‌شود. از مزایای آن می‌توان به کاهش احتمال خطرات سوختگی در نوروپاتی‌های شدید اشاره کرد. هدف از انجام این مطالعه بدست آوردن مقادیر طبیعی و بررسی تکرارپذیری این تست بعنوان مرجعی برای مقایسه مقادیر غیرطبیعی (محدوده طبیعی) در جامعه ایرانی می‌باشد. روش بررسی: این مطالعه به روش مقطعی و بصورت توصیفی در سه ماهه اول سال 1383 در کلینیک‌های وابسته به دانشگاه علوم پزشکی شیراز انجام گرفت. جهت بررسی مقادیر نرمال، 50 فرد سالم (32 مرد و 18 زن) با محدوده سنی 20 تا 40 سال که سابقه‌ای از مشکلات عصبی- عضلانی، ابتلا به دیابت و الکلیسم نداشتند بطور تصادفی با روش نمونه‌گیری ساده انتخاب شده و آزمایش آستانه درک جریان در درماتومهای 8C (انگشت چهارم دست) و 5L (انگشت شست پا) توسط دستگاه نورومتر (CPTc) بر روی آنها انجام گردید. جهت بررسی تکرارپذیری تست تعداد 6 نفر روزی 3 بار و در طول یک هفته 2 بار مورد آزمایش آستانه درک جریان قرار گرفتند. سپس داده‌ها جهت تعیین میانگین، انحراف معیار و تکرارپذیری تست آنالیز شدند. یافته‌ها: مقادیـر نرمال برای درماتوم 8C در فرکانسHz 2000 عبارت بود از 47± 2/04 میلی آمپر، در فرکانس Hz 250 ،0/25±0/75 میلی آمپر و در فرکانس Hz5  0/21±0/51 میلی آمپر و برای درماتوم 5L در فرکانس Hz 2000 ،0/73±2/83 میلی آمپر، در فرکانس Hz 250، 45/0± 24/1 میلی آمپر و در فرکانس Hz 5، 0/30±0/76 میلی آمپر. آزمون آلفای کرونباخ جهت بررسی قابلیت اعتماد و تکرارپذیری تست استفاده شد، قابلیت اعتماد در اندام فوقانــی (%98/5) و در اندام تحتانی 99% می‌باشد (0/9875=a1 و 0/9980=a2). نتیجه‌گیری: یافته‌های بدست آمده نزدیکی زیادی با مقادیر طبیعی جهانی دارد و می‌تواند بعنوان بخشی از بانک اطلاعاتی محدوده طبیعی ایران استفاده شود. ولی بررسی‌های بیشتر در این رابطه، بخصوص در کلینیکها و مراکز دیگر بر ارزش و اعتبار این بانک اطلاعاتی خواهد افزود، چراکه این داده فقط از درماتومهای 8C و 5L بدست آمده است. در ضمن تکرارپذیری تست رابطه مستقیم با میزان تبحر فرد آزمونگر و رعایت دقیق نکات مندرج در روش انجام تست دارد و بدین ترتیب قابلیت اعتبار تست در هر کلینیک باید بطور جداگانه مورد بررسی قرار گیرد.

Title: مقایسه‌ تأثیر فوری تکنیک تیپینگ و بریس کانترفورس بر روی درد و قدرت گیرش دست

http://rehabilitationj.uswr.ac.ir/article-1-59-fa.html

هدف: وجود درد و ضعف در قدرت گرفتن دست(Grip) اساسی ترین مشکل بیماران اپی کندیلیت خارجی می‌باشد. استفاده از اسپلینت یکی از راههای درمان این بیماران می‌باشد که باعث کاهش درد و افزایش قدرت گرفتن در این بیماران می‌شود. تکنیک تیپینگ (taping) و بریس کانترفورس (counterforce) دو روش مورد استفاده در درمان این بیماران می‌باشند ولی اینکه کدام یک از این دو روش مؤثرترند مدرک مستندی موجود نمی‌باشد. مقایسه تأثیر این دو روش در بهبودی بیماران اپی کندیلیت خارجی هدف این مطالعه می‌باشد. روش بررسی: این تحقیق به روش شبه تجربی (Quasi Experimental) انجام شده و جامعه مورد مطالعه شامل 30 بیمار (دو گروه 15 نفره) با تشخیص اپی کندیلیت خارجی و با گروه سنی 55-30 سال می‌باشند که به صورت موارد در دسترس انتخاب شده و به طور اتاقی به دو گروه تقسیم شدند و دو روش تیپینگ یا بریس کانترفورس در مورد آنها انجام شد. یافته‌ها: در تست قدرت گرفتن با توجه به اختلاف معنی داری بین دو روش مورد استفاده در گروه‌ها دیده نشد (0/06=P). بنابراین اختلاف قابل توجهی بین تکنیک تیپینگ و بریس کانترفورس بر میزان قدرت گریپ بیماران اپی‌کندیلیت خارجی وجود ندارد. در بررسی میزان درد در دو وضعیت استراحت و اکستنشن مچ دست بین دو روش اختلاف معنی‌داری وجود دارد و در هر دو وضعیت، میانگین نمرات گروه تیپینگ بیشتر از دو گروه کانترفورس است (0/001=P و 0/004=P). نتیجه‌گیری: با توجه به نتایج به دست آمده و اختلاف معنی‌دار دو روش در کاهش درد، تأثیر تکنیک تیپینگ بر کاهش درد بیماران بیشتر است و بهتر است از این روش استفاده شود ولی در افزایش قدرت گریپ با اینکه نمرات گروه تیپینگ بیشتر است ولی اختلاف دو روش معنی‌دار نیست و توصیه می‌شود بررسی مجددی با نمونه‌های بیشتر و سنجش دقیقتر انجام شود.

Title: بررسی تأثیر تکنیک‌های حفظ انرژی بر میزان خستگی بیماران زن مبتلا به مولتیپل اسکلروزیس

http://rehabilitationj.uswr.ac.ir/article-1-60-fa.html

هدف: بیماری مولتیپل‌اسکلروزیس جزء بیماری‏های شایع دمیلینیزاسیون سیستم عصبی مرکزی می‏باشد. عوارض ناشی از بیماری مولتیپل‏‏اسکلروزیس می‌تواند اثر مخربی بر ایفای نقش و وضعیت شغلی این بیماران و در نتیجه کیفیت زندگی آنان داشته باشد. از جمله این عوارض شایع خستگی است، لذا این پژوهش با هدف تعیین تأثیر به‌کارگیری تکنیک‌های حفظ انرژی بر میزان خستگی این مددجویان انجام شده است.  روش بررسی: نمونه‌های این پژوهش نیمه تجربی را سی و نه مددجوی زن مبتلا به بیماری مولتیپل‏‏اسکلروزیس به صورت نمونه‌های در دسترس تشکیل می‌دادند. مطالعه به‌صورت قبل و بعد و به‌مدت سه ماه انجام شد. ابزارهای جمع‌آوری اطلاعات شامل پرسشنامه اطلاعات دموگرافیک، پرسشنامه سنجش شدت خستگی و چک‌لیست‌های خودگزارش‌دهی و مداخله شامل دو قسمت بود: بخش اول آموزش و نمایش و بخش دوم آموزش رفتارهای حفظ انرژی که با روش سخنرانی به همراه پرسش و پاسخ و همچنین استفاده از پمفلت‌های آموزشی انجام شد. سپس خستگی سه مرحله، قبل از مداخله، یک‌ونیم ماه بعد از مداخله و سه ماه پس از مداخله بررسی و نیز همراه سایر اطلاعات حاصل از پرسشنامه‌ها مورد تجزیه و تحلیل و آنالیز واریانس قرار گرفت. یافته‌ها: میانگین خستگی‌ نمونه‌های پژوهش قبل از انجام مداخله 5/36 بود که پس از 1/5 ماه از انجام مداخله به 4/97 کاهش یافت و اختلاف این دو مرحله معنی دار می‌باشد (P>0/001). میانگین خستگی بعد از 3 ماه از انجام مداخله به 4/58 رسید که اختلاف بین مرحله قبل و 3 ماه بعد و مرحله 1/5 ماه و 3 ماه نیز با P>0/001 معنی دار می‌باشد. مقایسه میزان خستگی هر سه مرحله از طریق آنالیز واریانس نیز نشان دهنده اختلاف معنی دار می‌باشد (P>0/001). نتیجه‌گیری: با توجه به یافته‌های پژوهش می‌توان نتیجه گرفت که اجرای تکنیک‌های حفظ انرژی در مبتلایان به بیماری مولتیپل‌اسکلروزیس موجب کاهش میزان خستگی می‌شود. بنابراین بکارگیری این روش غیردارویی می‌تواند مفید بوده و به راحتی توسط بیماران مورد استفاده قرار گیرد.

Title: آنالیز پیوستگی 50 خانواده ایرانی مبتلا به ناشنوایی غیر سندرمی با وراثت اتوزومی مغلوب

http://rehabilitationj.uswr.ac.ir/article-1-61-fa.html

هدف: از هر 1000 نوزاد متولد شده در سراسر جهان یک نفر مبتلا به ناشنوایی بوده که 50% از علل آن ژنتیکی می‌باشد. ارتباط جایگاه ژنی DFNB21 با ناشنوایی غیر سندرمی جسمی مغلوب در کشورهای همسایه ایران در چندین مطالعه نشان داده شده است. بدین منظور 50 خانواده ایرانی مبتلا به ناشنوایی غیر سندرمی جسمی مغلوب برای آنالیز پیوستگی با این جایگاه ژنی در این مطالعه مورد بررسی قرار گرفتند. روش بررسی: در این مطالعه که به روش توصیفی مقطعی انجام گرفت 50 خانواده مبتلا به ناشنوایی با الگوی وراثتی اتوزومی مغلوب از سراسر ایران به صورت نمونه‌های در دسترس و شناخته شده بر اساس منفی بودن موتاسیونهای وابسته به GJB2 و GJB6، وجود حداقل سه فرزند ناشنوا و علاوه بر آن ازدواج خویشاوندی برای بررسی جایگاه ژنی DFNB21 انتخاب شدند. یافته‌ها: از بین این خانواده‌ها (4%) 2 خانواده الگوی پیوستگی به لوکوس DFNB21 را نشان دادند. بررسی ژن Tecta با روش تعیین توالی جهشهای delT266 و 9611 جفت باز را به صورت هموزیگوت در افراد مبتلای خانواده‌ها، مسؤول اختلال در ناشنوایی نشان داد. نتیجه‌گیری: بنظر می‌رسد که جایگاه ژنی D‏FNB21 نقش قابل توجهی را در ایجاد ناشنوایی در جمعیت ایرانی ایفا کند. این موضوع می‌تواند با غربالگری خانواده‌های بیشتر برای این لوکوس بررسی شود.

Title: ارزش سنجش سطح بزاقی لیتیم در پایش درمان اختلالات خلقی

http://rehabilitationj.uswr.ac.ir/article-1-62-fa.html

مشتقات لیتیم از قرن نوزدهم جهت درمان بیماریها بخصوص اختلالات دو قطبی مورد مصرف قرار گرفته است. مسمومیت کشنده لیتیم از همان شروع مصرف آن شناخته شده بود و بعلاوه پایین بودن شاخص درمانی آن از مشکلات مهم در تجویز آن می‌باشد. بمنظور پیش‌گیری از مسمومیت فوق پایش سطح دارو در خون مورد توافق قرار گرفته است. خونگیری مکرر و بخصوص در دور تثبیت بصورت هفتگی برای بیماران توأم با استرس بوده و بعلاوه در بیماران روانی و بویژه در اطفال مشکل می‌باشد. از این رو محققین همواره در تلاش برای یافتن راهی کمتر تهاجمی بوده‌اند. یکی از این راهها پایش دارو در بزاق می‌باشد ولی بررسی‌ها بیانگر اختلاف نظر راجع به ارزش بزاق در پایش فوق می‌باشد. در این مقاله کلیه مطالعات منتشر شده از 1949 تاکنون مورد بررسی قرار گرفت و اختلاف نظر فوق بخوبی آشکار گردید. با توجه به بررسی فوق سه راه برای رفع این اختلاف نظر پیشنهاد گردیده است. 1- محاسبه نسبت غلظت سرمی لیتیم به غلظت بزاقی آن بر اساس متوسط سه جفت نمونه مختلف 2- بهبود روش و تکنیک آزمایشات و 3- اصلاح نسبت یاد شده بر اساس نشانگرهای طبیعی مشخص.

Title: گزارش دو مورد سندرم شیوگرن-لارسون از یک خانواده

http://rehabilitationj.uswr.ac.ir/article-1-63-fa.html

سندرم شیوگرن – لارسون یک اختلال اتوزومال مغلوب می‌باشد که مشخصه بیماری به صورت ایکتیوز شدید منتشر، عقب‌ماندگی ذهنی، دی‌پلژی یا تتراپلژی اسپاستیک و تشنج می‌باشد. این اختلال از سندرم‌های نادر بوده و در اثر جهش در ژن 2A3ALDH اتفاق می‌افتد. این ژن بر روی کروموزوم 17p11.2 قرار داشته و عمل آن کد نمودن آنزیم «فتی آلدئید دهیدروژناز» است. این آنزیم اکسیداسیون آلدئیدهای با زنجیره متوسط و بلند از الکل چرب را کاتالیز می‌نماید. یافته‌های تصویر برداری مغز در این بیماران گویای تأخیر میلینیزاسیون و نقص میلین است که با ام.آر.آی قابل مشاهده است. در بررسی‌های به عمل آمده توسط ام.آر.اس (ماگنتیک رزونانس اسپکتروسکوپی) در بافت سفید مغزی پیک غالبی از تجمع لیپید که همان فتی آلدئیدها و فتی الکل‌ها می‌باشد قابل مشاهده است. بیماران معرفی شده دو خواهر و برادر از والدین منسوب نزدیک است که تنها فرزندان خانواده می‌باشند. برادر 6 ساله و خواهر 3 ساله و هر دو مبتلا به تأخیر تکاملی، عقب‌ماندگی ذهنی، اسپاستیسیتی شدید اندام‌های فوقانی و تحتانی و تشنج مقاوم به درمان هستند. مسئله قابل توجه وجود ایکتیوز شدید منتشر پوستی در هر دو بیمار می‌باشد. در بررسی‌های به عمل آمده از لحاظ تصویر برداری مغزی در ام.آر.آی تغییر سیگنال بافت سفید مغزی و در ام.آر.اس پیک قابل ملاحظه لیپید مشاهده گردید که تشخیص سندرم «شیوگرن – لارسون» را تأیید نمود.
